# Selection of Roasting Conditions in the Valorization Process of Cornelian Cherry Stones

**DOI:** 10.3390/molecules30142900

**Published:** 2025-07-08

**Authors:** Radosław Spychaj, Dominika Przybylska, Małgorzata Szachniewicz, Narcyz Piórecki, Alicja Z. Kucharska

**Affiliations:** 1 Department of Fermentation and Cereal Technology, Wrocław University of Environmental and Life Sciences, 51-630 Wrocław, Poland; maszach@gmail.com; 2 Department of Fruit, Vegetable and Plant Nutraceutical Technology, Wrocław University of Environmental and Life Sciences, 51-630 Wrocław, Poland; dominika.przybylska@upwr.edu.pl (D.P.); alicja.kucharska@upwr.edu.pl (A.Z.K.); 3 Arboretum and Institute of Physiography in Bolestraszyce, 37-722 Przemyśl, Poland; npiorecki@ur.edu.pl; 4 Institute of Physical Culture Sciences, Medical College, University of Rzeszów, 35-959 Rzeszów, Poland

**Keywords:** *Cornus mas* L., stone, roasting parameters, bioactive compounds, iridoids, antioxidant capacity, solvent extraction

## Abstract

The aim of the study was to assess the effect of time (20–50 min) and temperature (160–220 °C) of roasting on the physicochemical and antioxidant properties of cornelian cherry (*Cornus mas* L.) stones and to select extraction solvents (water and 50% and 80% methanol) to obtain reliable results. To maintain the highest content of bioactive compounds with a high level of antioxidant properties, roasting at a temperature of 160–180 °C for 30–40 min should be considered optimal. Incorrect selection of roasting parameters (>40 min and >200 °C) causes a significant reduction in the bioactive properties of roasted stones. Regression analysis showed a different nature of changes in the determined features during the roasting process at 160 °C than at other temperatures. The use of methanol, especially at a high concentration (80%), to assess the content of bioactive compounds in roasted cornelian cherry stones provides more reliable results.

## 1. Introduction

The genus *Cornus* (*Cornaceae*) comprises several dozen species, described as trees or shrubs, occurring mainly in the Northern Hemisphere and temperate climates [1,2,3,4]. The vast majority of these plants are ornamental in value and can be commonly found in parks and gardens owing to their flowering period, the form and color of inflorescences, winter coloration of stems into purple-carmine, and diversified color of fruits [2,5]. However, due to the flavor qualities and unique chemical composition, the fruits of certain species are also used in the food industry, for example, for the production of jams, juices, liqueurs, and distillates (*Cornus mas* L.), or for the production of supplements or nutraceuticals in the pharmaceutical industry (*Cornus officinalis* Sieb. et Zucc.) [4]. These fruits are considered valuable because of their high concentrations of vitamins (vitamin C in particular), minerals, and the presence of secondary metabolites, such as iridoids, anthocyanins, phenolic acids, or flavonols [1,4,5]. Recently, multiple articles have been published concerning the biological properties of the European species *C. mas*. The presence of bioactive compounds in *C. mas* makes the fruit and the preserves obtained therefrom possess beneficial effects for the cardiovascular system and have hypolipidemic, anti-diabetic, anti-inflammatory, or anti-amnesic effects [4,6,7]. At the same time, the interest in cornelian cherries (*C. mas*) is growing, particularly in the food industry, which contributes to generating substantial amounts of by-products resulting from the share of the stone in the fruit, amounting to >20% [8,9]. In addition to the main product, the processing of this raw material into preserves generates by-products, such as pomace from stones in juice production or solely stones in jam production. The residual parts of cornelian cherries, depending on the initial raw material, technology, and processing conditions, can be valuable due to the concentrations of certain compounds. Cornelian cherry stones contain more dry weight, minerals, and unsaturated fatty acids than the fruit flesh does [1,5,10].

The chemical composition of *Cornus mas* stones is scarcely described in the literature. According to Antoniewska-Krzeska et al. [5] (2022), the dry weight of cornelian cherry stones accounts for > 90%, the protein content for 2.27%, and fat for 3.4%. Other authors report a wider range of fat content in cornelian cherry stones, i.e., from 2.9 to 6.1%, depending on the cultivar [1,10]. According to Kucharska [1] (2012), the content of fat in the stone is over fivefold greater than that of the flesh (2.9% and 0.51%, respectively). The analysis of the fatty acid composition showed that linoleic acid predominated in the lipid fraction of stones, with its share in the total lipid fraction ranging from 60.18% to 73.99%, depending on the cultivar. Among the remaining fatty acids of this fraction, the presence of oleic and palmitic acid has been demonstrated to represent up to 10% [1,5,10,11]. Virdih et al. [10] reported similar amounts of linoleic and palmitic acid (68.23% and 7.71%, respectively). However, in samples tested by the authors, the share of oleic acid in the total fatty acid content ranged from 15.5% to 22.9%. The compounds of Ca, K, and P are among the most abundant minerals in cornelian cherry stones [5,10]. According to Spychaj et al. [12], cornelian cherry stones, as well as the fruits, contain bioactive compounds, such as iridoids (loganic acid (LA), cornuside) or polyphenols (ellagic acid (EA)). Moreover, in *C. mas* stones, Przybylska et al. [13] identified 37 compounds belonging to the groups of gallotannins and ellagitannins. This raw material also demonstrates significant antioxidative potential [12,13].

The properties of fruit stones and their valorization are diverse and depend primarily on the raw material from which they are derived [14]. Matos et al. [15], Haimour & Emeish [16], and Aygün et al. [17] report that the stones of olives and apricots or date palm seeds can be used in the production of activated carbon. The presence of various classes of bioactive compounds in fruit stones should be considered an advantage; however, in the case of cherry, peach, apricot, or plum stones, cyanogenic glycosides such as prunasin and amygdalin have been found, which can complicate their application in food production. The presence of these compounds has not been reported in olive stones, date palm fruits, or cornelian cherry stones [1,14,18]. The seeds of date palms contain 8.5–10.4% fat, with the highest contributions to the fat content belonging to oleic acid (31.8–50.0%) and lauric (10.2–30.8%) [19,20,21]. According to Padilla-Rascón et al. [22] (2020), proper treatment of olive stones with the use of steam at temperatures of 180–225 °C can yield high recovery of glucose or xylose. Ordoudi et al. [14] state that the extracts from olive stones, obtained with the use of polar solvents, are rich in phenolic compounds, while the use of non-polar solvents allows the generation of oil rich in sterols, tocopherols, tocotrienols, or squalene. The stones of olives used as combustible material demonstrate a heating value of approximately 17 MJ/kg [15,23]. They are also used as a raw material in the production of activated carbon or bioethanol [23] (García Martín et al. 2020). However, most valorization methods refer to date palm seeds, which stems from the availability of this raw material and its high contents of fat, dietary fiber, phenolics, and antioxidative potential [24]. The production of oil with a valuable composition of fatty acids is a very popular method of valorization for date seeds [19,20]. Numerous studies have been conducted related to the roasting of fruit stones and the application of unroasted stones as additives in the production of bakery products, meat, and beverages that are coffee substitutes [24,25,26]. In the case of date seeds, their proposed application involves the roasting process within the range of 160–220 °C for 10–30 min [21,24,27] after seed defatting [28,29]. High amounts of bioactive compounds and transformations of lipids and volatile compounds occurring in roasting make brews from roasted dates similar to brews from grain coffee in taste and aroma [10,21,25]. In addition, Fikry et al. [27,28] noted that intensification of the roasting process was accompanied by an increase in the TPC and the antioxidative potential of brews from roasted date seeds.

The stones, apart from other morphological parts of cornelian cherries, were used in Turkey for the preparation of traditional medicines [30]. Scientific reports regarding the potential nutritional application of cornelian cherry stones were presented by Dupak et al. [31], who noted a significant drop in the blood glucose levels of ZDF rats after oral gavage of crushed cornelian cherry stones. The animals received a dose of 250 mg/kg rat bw (body weight) of cornelian cherry stones for 14 weeks. After the treatment, the glucose level decreased from 18.83 mmol/L (control group) to 9.83 mmol/L (experimental group). The use of cornelian cherry stones in bio-oil production is also suggested as a possible valorization of this raw material [32,33]. The roasting of cornelian cherry stones, however, appears to be a valuable valorization method [12]. The stones roasted at 170 °C for 30 min are still characterized by considerable antioxidative potential and frequently contain higher contents of bioactive compounds (loganic acid, ellagic acid, and its derivatives) than the stones before roasting [12]. At the same time, the authors observed the formation of 58 volatile compounds during the roasting process, belonging mainly to derivatives of pyrazines, contributing to the development of aromas described as nutty, coffee, or chocolate flavor. Despite this, the available literature lacks information on the influence of roasting conditions of *Cornus mas* in a broader range of temperatures and times on the content of bioactive compounds and the antioxidative potential of roasted stones.

In view of the above, the aim of the present study was to evaluate the influence of time and temperature on the roasting of cornelian cherry stones (*Cornus mas*) for quality attributes, the contents of individual bioactive compounds, and the in vitro antioxidant capacity of roasted stones. The effects of solvents on the extraction of compounds from cornelian cherry stones and the antioxidant capacity of the obtained extracts were also evaluated.

## 2. Results and Discussion

### 2.1. Evaluation of the Roasting Effect on the Physical Properties of the Stones

**Figure 1:**
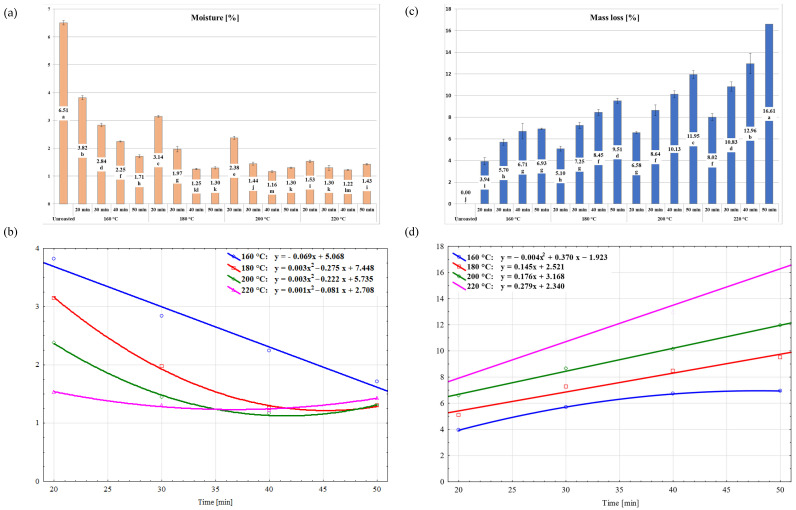
Moisture content (**a**) and mass loss (**c**) of unroasted and roasted cornelian cherry stones (*Cornus mas* L.) and their changes (**b**,**d**) during roasting.

**Figure 2:**
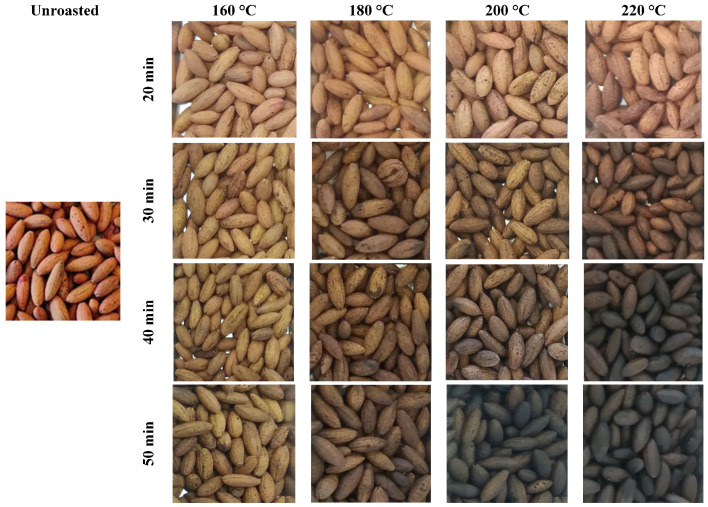
Unroasted and roasted cornelian cherry stones (*Cornus mas* L.).

**Figure 3:**
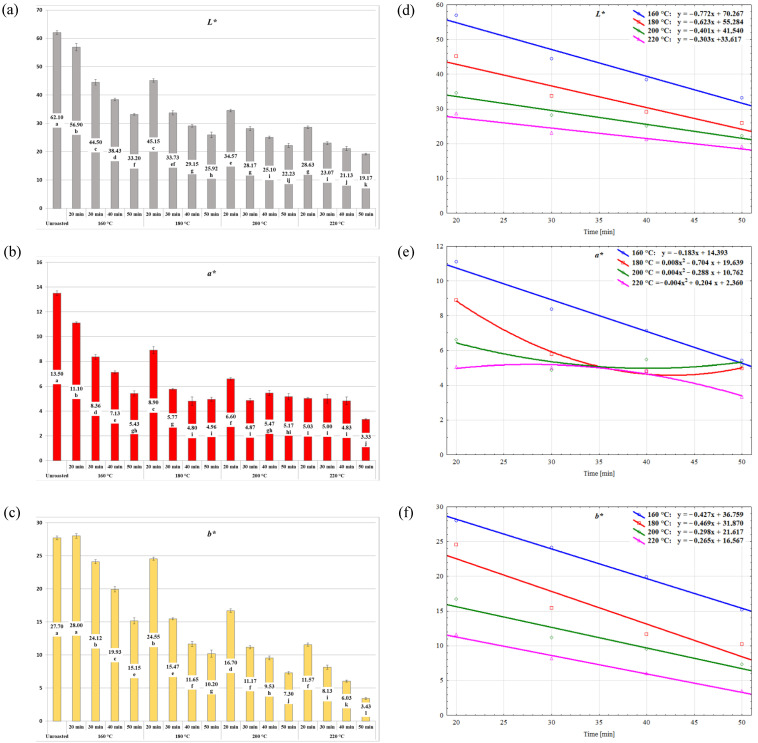
Color characteristics (L*a*b*) (**a**–**c**) of unroasted and roasted cornelian cherry stones (*Cornus mas* L.) and their changes (**d**–**f**) during roasting.

**Figure 4:**
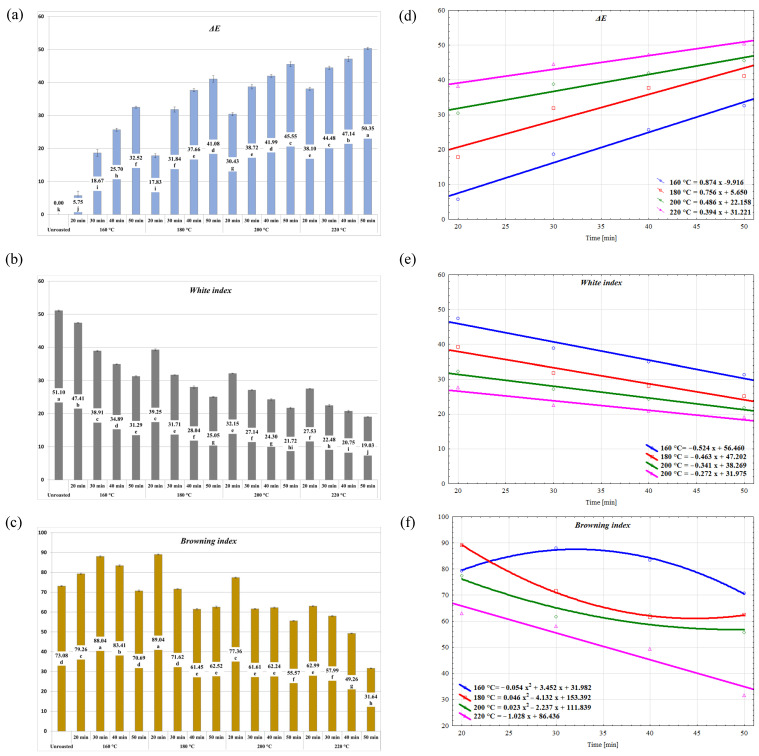
Color characteristics of unroasted and roasted cornelian cherry stones’ (*Cornus mas* L) ΔE (**a**), white index (**b**), browning index (**c**), and their changes during roasting (**d**–**f**).

**Figure 5:**
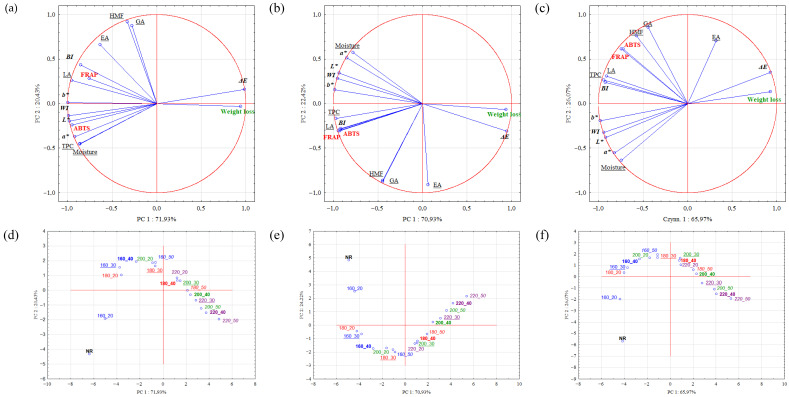
Principal component analysis of cornelian cherry (*Cornus mas* L.) stones for individual solvents (water: (**a**,**d**); 50% methanol: (**b**,**e**); and 80% methanol: (**c**,**f**)).

The analysis of the physical properties of cornelian cherry stones included moisture, roasting weight loss, and color parameters in the CIE L*a*b* system. The results are illustrated in Figure 1, Figure 2, Figure 3, Figure 4 and Figure 5.

The moisture of unroasted cornelian cherry stones amounted to 6.51% (Figure 1a). Roasting caused water evaporation, and its degree depended on the temperature and time of the process. The roasted stones contained between 1.16% (200 °C; 40 min) and 3.82% (160 °C; 20 min) of water. A decrease in moisture is accompanied by an increase in stone tenderness, which is a desirable feature for roasted products. The moisture of cornelian cherry stones roasted for 20 min at 160 °C decreased by approx. 40%, while at 220 °C the moisture decreased by nearly 80%. Longer roasting times increased the degree of changes in the moisture content, and the highest—over 82%—was observed for roasting at 200 °C for 40 min. The increase in the duration of roasting at different temperatures resulted in different dynamics of changes in the moisture content of the stones (Figure 1b). The dynamics of changes in moisture content could be described by a linear function (y = −0.069x + 5.068) only at the temperature of 160 °C. At the remaining roasting temperatures, the changes in moisture were described by a quadratic function determined by the equations shown in Figure 1a. Fikry et al. [28] determined a linear relation between date seed moisture at the applied roasting temperatures (160–200 °C) and roasting times (10–30 min) when considering the roasting time, and as a quadratic function when considering the influence of temperature and its interaction with the roasting time. Babiker et al. [21] obtained different observations. According to the authors, roasting of date seeds for 20 min at the temperatures of 180, 200, and 220 °C did not cause a differentiation between samples in terms of moisture content, which ranged from 3.58 to 3.90%. The variation in the degree of moisture changes between cornelian cherry stones and date seeds with short roasting times could be due to different chemical compositions of the stones and the seeds, principally distinct contents of the oil fraction, and distinct structures of each material [1,21].

The changes in weight loss during roasting followed a different pattern compared to moisture (Figure 1c). It was found that the rate of weight loss increased with the increase in roasting time and temperature, but with different dependency (Figure 1d). Longer roasting times resulted in a linear increase in weight loss at 180 °C, 200 °C, and 220 °C. The dynamics of changes in this attribute were described by a quadratic function (y = −0.004x^2^ + 0.370x + 1.923) only at the temperature of 160 °C (Figure 1d). The losses observed at the lowest temperature reached the highest level of 6.71% after 40 min of the process, and did not increase significantly at longer roasting times. It can be concluded that after 20 min of roasting, the loss reached half the level reported at the longest roasting time. According to Ghnimi et al. [25], the greatest weight loss during roasting of date seeds takes place at the initial phase of the process, which is related to the removal of water and compounds with low evaporation temperatures, e.g., volatiles. Cho et al. [34] reported that weight loss during roasting is tightly linked to the roasting degree of Arabica coffee beans (*Coffee arabica* L.) and results in 10.00% weight loss for a “light” roast lasting 9 min at 240 °C, but for a “very dark” roast, obtained after 16-min roasting at the same temperature, the loss amounts to 21.74%. Cämmerer & Kroh [35] state that roasting a blend of Arabica and Robusta coffee beans (80:20) in industrial conditions generates weight loss ranging between 14.5 and 18.9%, depending on the roasting degree.

The unroasted stones were characterized by the L*a*b* color parameters of 62.1, 13.5, and 27.7, respectively, which can be described as gray and pink-yellow (Figure 2 and Figure 3a–c). Roasting at the mildest conditions (160 °C; 20 min) gave a product color described by the following L*a*b* parameters: 56.90, 11.10, and 28.00, respectively. The stones roasted at 220 °C for 50 min were characterized by a color described by the L*a*b* parameters of 19.17, 3.33, and 3.43, respectively. The obtained parameters indicate the color change toward black and the fading of red hue. It was also reported that the roasting times applied in this work yielded products that were significantly different in terms of color parameters L* and b* within each temperature (Figure 3a,c). For the a* parameter, this trend was observed only at 160 °C (Figure 3b).

The above-mentioned observation was explained in detail by the regression equations shown in Figure 3d–f. The performed regression analysis allows the conclusion that the changes in parameters L* and b* follow a linear trend at each of the applied temperatures depending on the roasting time. Moreover, the slope coefficients in regression equations of the L* parameters take negative values for individual temperatures: −0.772, −0.623, −0.401, and −0.303, respectively, which reflect various rates of change of this parameter at the applied roasting times. The regression slope coefficients of the b* parameters for roasting at 160, 180, 200, and 220 °C were at similar levels: −0.427, −0.469, −0.298, and −0.265, respectively. It indicates a smaller influence of the roasting temperature on the differentiation of change dynamics of the b* parameter than of the L* parameter. For the a* parameter, a linear pattern of change was noted only at 160 °C (y = −0.183x + 14.393). The changes in this color parameter at the remaining temperatures were described by quadratic functions.

Figure 4a–c show the values of the *ΔE*, *WI*, and *BI* parameters for the individual experimental objects and the nature of their changes (Figure 4d–f, Table S1) with roasting times at individual temperatures. The calculated parameters show a linear nature of changes with roasting time in the case of the *ΔE* and *WI* parameters (Figure 4d–e, Table S1). The recorded changes of the *BI* discriminant value during roasting were linear only in the case of roasting cornelian cherry stones at 220 °C (Figure 4f, Table S1).

Evaluation of the advancement of the roasting process is based on the selection of the roasting time at a set temperature and a visual observation of, for example, color changes of roasted material resulting from the progressing Maillard reactions [24]. The study by Wong et al. [36] showed a relation between low moisture and intensity of color changes. It is related to obtaining products characterized by a darker color at shorter times and higher temperatures and during the final stages of roasting. Visible changes of color are the first results of roasting, and significant extension of the process does not provoke as big of changes in color as in the first phase [21,25]. In addition to the linear changes in color, the intensification of roasting date stones at 160, 180, and 200 °C for 30 min caused a similar trend of hardness reduction of the final product, but only at the lowest roasting temperature, 160 °C [24]. In the case of roasting Robusta coffee beans at varying process temperatures, ranging from 160 to 225 °C, it was shown that the final color of the roasted product, developed during roasting, is formed after cooling and can also change during storage, especially for the L* parameter [37]. In the case of roasting cornelian cherry stones discussed in this paper, color changes occurred throughout the process, and their intensity was higher at higher process temperatures. The linear characteristics of changes in the L* and b* parameters allow for the conclusion that visual evaluation of the advancement of roasting is possible in the case of cornelian cherry stones.

### 2.2. The Contents of Bioactive Compounds and HMF by the HPLC-PDA Method

The contents of loganic acid (LA), gallic acid (GA), and ellagic acid (EA) in the raw and roasted stones were determined in aqueous and 50% and 80% methanolic extracts. The results are presented in Table 1, and the dynamics of the changes are described by the regression equations included in Table S2.

In the unroasted stones, the contents of LA and EA were at different levels depending on the solvent used. The LA content was significantly higher in the aqueous extract (185.44 mg/100 g dw) than in the methanolic extracts (120.84 mg/100 g dw), whereas the EA content was higher in the 80% methanolic extract (362.73 mg/100 g dw) than in the aqueous extract (204.32 mg/100 g dw). The content of GA, however, did not differ significantly among the studied extracts. The contents of LA and GA in the aqueous extracts from unroasted stones were, respectively, from more than 1.5 to 3 times, and from 60 to 185 times higher than in our previous research, where we studied freshly separated cornelian cherry stones (no thermal treatments applied during fruit processing) using hot water extraction (Spychaj et al. 2021 [12]). Such large discrepancies, especially in GA levels, were probably a result of using technological stones in the earlier study, in which stones underwent a thermal treatment before being separated from the ripe fruits. The thermal treatment may have caused the hydrolysis of tannins, which occur in cornelian cherry fruits [38] and stones [13], and the migration of these compounds from the flesh of the fruit to the stone. In addition, in the previous study we used ultrasound-assisted extraction, which can also cause the hydrolysis of polymeric tannins to monomeric forms, such as EA and GA.

After the roasting process, the extraction of the analyzed compounds differed depending on the solvent used, but significantly higher amounts of all acids studied were reported in the 80% methanolic extracts, followed by the 50% methanolic extracts and aqueous extracts.

Compared to unroasted stones, higher contents of LA were observed at lower temperatures and shorter times, specifically after methanolic extraction. It suggests that mild roasting conditions caused the release of LA from high-molecular structures and better extraction of this compound from the roasted material, especially in 80% methanolic extraction, while under the conditions of 180 °C and 40 min, 200 °C and 30 min, and 220 °C and 20 min, LA gradually degraded up to 98% (aqueous extract, 220 °C, 50 min).

During roasting, GA and EA were released from the structures of hydrolyzable tannins [12]. Maximum concentrations were determined in methanolic extracts from stones roasted under the conditions of 160 °C, 50 min; 180 °C, 30 min; and 200 °C, 20 min for GA and under the conditions of 180 °C, 50 min and 200 °C, 30 min for EA. In the case of GA, an 8-fold increase in its concentration took place after using the temperature of 160 °C for 50 min compared to unroasted stones, while in the case of EA a 32-fold increase was observed (180 °C, 50 min; 200 °C, 30 min). In our previous study we recorded an increase in EA content ranging from 3- to 32-fold in the stones roasted at 170 °C for 30 min compared to unroasted stones [12].

The characteristics of changes in concentrations of the studied compounds during roasting at the individual temperatures are presented by the regression equations along with the determination coefficients R^2^ (Table S2). In the case of LA, the regression equations determined for the temperatures of 180, 200, and 220 °C followed a linear model, regardless of the solvent used. However, for roasting at 160 °C, the regression equations showed low values of the coefficient of determination R^2^ (0.002–0.130), indicating low association/fitting of the observed changes to a linear function. The determination coefficients for the quadratic function were within a range of 0.940–0.986. As in the case of LA, the changes in GA content followed a linear model for most samples. However, for roasting at 180 °C, the characteristics of changes were better described by a quadratic function.

In the study by Samsonowicz et al. [39], coffee substitutes from raw materials of different origins did not contain GA and EA, or contained considerably high concentrations, e.g., acorn coffee with ginseng with values of 1665.8 mg/kg and 2734.9 mg/kg, respectively. From this perspective, the possibility of application of roasted cornelian cherry stones as an additive to grain coffee substitutes appears reasonable given the characteristics of volatile aroma compounds of roasted cornelian cherry stones [12].

In the unroasted stones, the presence of HMF was not reported, irrespective of the solvent used (Table 1). However, HMF was formed as a result of the reactions taking place at high temperatures and was observed in significantly higher amounts in the methanolic extracts (203.47–214.05 mg/100 g dw) than in the aqueous extracts (181.36 mg/100 g dw). The concentration of HMF gradually increased at all the applied roasting temperatures, reaching maximum concentrations after 20 min (200 °C and 220 °C) and 30 min (160 °C and 180 °C), and then decreased until its complete degradation. In the aqueous extract, HMF was not reported for the roasting of the stones at 220 °C for 50 min, and in the 80% methanolic extract at 200 °C and 220 °C similarly for 50 min. According to Murkovic & Bornik [40] (2007), during the roasting of Robusta coffee beans at 240 °C, the highest amounts of HMF and its derivative HMFA (5-hydroxymethyl-2-furoic acid) are formed after approximately 3 min. The authors also concluded that coffees with higher roasting degrees did not contain this compound, which is in line with our results. In roasted and ground coffee, HMF can occur in a wide range of 300–1900 mg/kg, and in the fruit of date palms it can amount to 1000 mg/kg [41]. The results presented by the aforementioned authors were comparable to our results obtained for roasted cornelian cherry stones.

The analysis of regression showed that the change in HMF content during roasting at 180, 200, and 220 °C followed a linear pattern (Table S2), while at 160 °C, as in the case of LA, it fit a quadratic function.

### 2.3. Determination of Total Phenolic Content (TPC) and Antioxidant Capacity

The total phenolic content (TPC) and antioxidant capacity as determined by the ABTS and FRAP methods are presented in Table 2. The dynamics of changes in the discussed attributes are presented by the regression equations in Table S3.

No statistically significant differences were found in the TPC, the antioxidant capacity determined by ABTS, or the reducing power of Fe (III) ions by FRAP for the tested samples of unroasted stones (Table 2). After roasting, significant differences were observed between all extracts. The values of the TPC, ABTS, and FRAP obtained for 80% methanolic extracts were higher than those of the 50% methanolic and the aqueous extracts. In the case of 80% methanolic extraction, high levels of the TPC, ABTS, and FRAP were noted for the stones roasted at 160 °C for longer times (30–40 min). The aqueous extracts of stones roasted at this temperature were also characterized by high values of these attributes for a roasting time of 20 min. Similar levels of these attributes also were obtained for methanolic extracts of the stones roasted for a similar time at 180 °C. An increase in the roasting temperature to 200 °C and 220 °C for 50 min and 40–50 min, respectively, resulted in extracts with low values of the TPC, ABTS, and FRAP.

The characteristics of changes in the discussed attributes fit a linear model for most of the applied temperatures and for most of the extracts (Table S3). The changes in the TPC value followed a linear model for all the temperatures and the solvents applied. A distinct character of changes was observed for the antioxidant potential (ABTS) of 50% methanolic extracts and for the reducing power of ferric ion Fe (III) (FRAP) of both types of methanolic extracts from the stones roasted at 160 °C.

The antioxidative potential and the TPC of roasted cornelian cherry stones were higher than those of unroasted stones when the solvent used was 50% or 80% methanol. A significantly higher TPC and antioxidative potential of the studied *C. mas* fruits were reported when extraction was carried out with organic solvents, such as acetone, methanol, ethanol, or acetonitrile [42]. According to Spychaj et al. [12] (2021), the aqueous extracts from roasted cornelian cherry stones had lower antioxidative potentials (ABTS, FRAP) and TPCs than the extracts from unroasted stones, independent of the cultivar. The differences of the antioxidant activity among individual extracts from roasted stones and between the individual types of extracts should be linked to the transformations of tannins present in unroasted cornelian cherry stones [13] (Przybylska et al. 2020).

Cho et al. [34] (2014) reported that roasting of *Coffee arabica* L. beans in mild conditions (9 min, 240 °C) results in higher TPC and antioxidative potential of the brews prepared from it than from unroasted coffee. Cämmerer & Kroh [35] also point to a higher antioxidative potential of brews prepared from roasted coffee than unroasted coffee even compared to “medium” roast coffee grains.

### 2.4. Principal Component Analysis (PCA)

The PCA analysis was carried out separately for each of the solvents used (water, 50% methanol, and 80% methanol) for the assessed attributes, comparing experimental objects; the results are presented in Figure 5a–f. The total contribution of the influence of the principal components PC1 and PC2 ranged from 89.04 (80% methanol) to 93.35 (50% methanol), which indicates that the determined principal components illustrate a very high degree of explanation of the relationships of the studied attributes in all extracts.

The correlation of the moisture of roasted samples, *ΔE*, and of the weight loss during roasting with LA content showed significant similarity regardless of the solvent used (Figure 5a–c). The content of EA was negatively correlated with the stones’ moisture in the case of methanolic extracts, and likewise with the weight loss during roasting for aqueous extracts. Regardless of the solvent used, a significant positive correlation between HMF and GA contents was observed (Figure 5a–c). For both methanolic extracts, a strong positive correlation between LA and TPC contents was observed. In the case of aqueous extracts, this correlation was markedly weaker but still significant. Strong correlations between HMF content and ABTS and FRAP were found only for the methanolic extracts.

The PCA analysis performed allowed us to compare the unroasted stones to the stones roasted under different conditions, the results of which are presented in Figure 5d–f. It can be concluded that the distribution of samples on the PCA diagram shows significant similarity for all the applied solvents; however, for 50% methanol, their distribution in relation to the principal components axis was distinct from the remaining solvents. The objects showed similar tendencies for all solvents. The unroasted stones (NR) and the stones roasted under the mildest conditions (160 °C; 20 min) were significantly different from the rest of the objects. This distribution of the samples demonstrates that solely the process of roasting affected the stones, and roasting at 160 °C for 20 min contributed to their slight modification regarding the evaluated characteristics. Two groups of significant similarity can be distinguished from the remaining stones (Figure 5d–f). The first group showing similar parameters includes stones roasted at 160 °C for 50 min, 180 °C for 30 min, and 200 °C for 20 min. The second group of similar stones includes those roasted at 180 °C for 40 min, 200 °C for 30 min, and 220 °C for 20 min. The obtained groups suggest that similar roasted stones can be obtained using different time–temperature process parameters.

The different structures and chemical compositions of biological materials suggest the selection of an adequate solvent for the extraction. Stankovic et al. [43] applied water, methanol, acetone, ethyl acetate, and petroleum ether as solvents for the extraction of compounds from cornelian cherry leaves, flowers, and fruits. In the individual anatomical parts of cornelian cherries, large amounts of the extracted phenolic compounds were observed with the use of different solvents. The aqueous extraction gave higher amounts for leaves, the methanolic extraction for inflorescences, and the extraction with ethyl acetate for fruits. Gillani et al. [44] demonstrated the influence of the solvent (water and 50% and 100% ethanol) and sonication during extraction on the content of phenolics and the antioxidative potential of the extracts from cornelian cherry (*Cornus mas*) fruits. In the study by Karaaslan et al. [42], among the solvents used (water, methanol, ethanol, acetone, and acetonitrile) to investigate cornelian cherry fruits by evaluating ABTS and TPC, the highest values were obtained after extraction with methanol and acetone, respectively.

## 3. Materials and Methods

### 3.1. Materials

The stones assessed in the study originated from ripe cornelian cherry fruits (*Cornus mas*) harvested in 2016 in the Arboretum in Bolestraszyce and constituted a technological waste from the production of jam obtained at the Department of Fruit, Vegetable and Plant Nutraceutical Technology. The pretreatment of fruits included a heating process (95 °C for 2 min) and, after cooling, the separation of stones from the flesh and skin of the fruit.

The samples of stones (60 g) were subjected to roasting for 20, 30, 40, and 50 min at the temperatures of 160 °C, 180 °C, 200 °C, and 220 °C using a bakery oven chamber (Ibis, GT 800) to generate 16 experimental objects, which were further analyzed. The roasting of samples was performed in three parallel repetitions, and afterwards, the stones roasted in all repetitions were combined. The raw stones were also examined.

### 3.2. Methods

#### 3.2.1. Chemicals and Reagents

The following reagents were used in this work: distilled water, methanol (50 *v*/*v* and 80 *v*/*v*), 2,2ʹazynobis(3-etylobenzotiazoline-6-sulfonic acid (ABTS), 6-hydroxy-2,5,7,8-tetramethylchroman-2-carboxylic acid (Trolox), 2,4,6-tri(2-pyridyl)-s-triazine (TPTZ), FeCl_3_, methanol, acetonitrile (gradient grade for HPLC), and formic acid (98–100%; SKU 27001-1L-M), which were acquired from Sigma (Germany, Schnelldorf). Iridoids and phenolic acids standards were purchased from Extrasynthese (France, Genay) and HMF was purchased from Merck (Germany, Darmstadt). All reagents were of analytical or HPLC grade.

#### 3.2.2. Sample Preparation

The cornelian cherry fruit was processed immediately after harvest. The waste in the form of stones without pulp and skins was dried at 20 °C (for about a week and mixed daily) to a constant mass and then roasted. The whole unroasted stones and the cooled roasted stones were vacuum packed and stored for further analysis at −18 °C. Unroasted and roasted cornelian cherry stones (20 g) were ground in a WŻ-1 grinder (1000 W, 6000 rpm, ~20 °C, 10 s) (ZBPP, Bydgszcz, Poland) and subjected to three separate extraction processes with the use of distilled water (H_2_O) and aqueous solutions of 50% and 80% methanol (*v*/*v*). The sample-to-solvent ratio was 1:10. The samples were extracted for 24 h and sonicated (10 min) twice (Polsonic, Warszawa, Poland), immediately after adding the solvent to the sample and before the separation of the solid phase from the mixture by centrifugation (3500 rpm, 10 min) (MPW-350).

#### 3.2.3. Evaluation of the Roasting Process

The weight loss (%) generated in the roasting process was described based on the weight of the stones before and after roasting. The moisture content in ground samples was determined using a Radwag MAX 50/1/NP moisture analyzer.

The color parameters L*a*b* were determined in freshly ground samples using a Konica Minolta CR 410 colorimeter with reference to the CIE L*a*b* color space system. As a summary, the color changes calculated were *ΔE*, the white index (WI), and the browning index (BI), which were calculated based on the determined L*a*b* parameters obtained for the 16 types of crushed and roasted cornelian cherry stones. The L*a*b* parameters obtained for crushed, unroasted stones were used as a reference point. The following formulas were used for calculations of the aforementioned parameters:

$$\Delta E=\sqrt{{\Delta L}^{2}+{\Delta a}^{2}+{\Delta b}^{2}}\;WI=100-\sqrt{({100-L)}^{2}+a^{2}+b^{2}}\;BI=\frac{100\;(x-0.31)}{0.17}\;\mathrm{where}\;x=\frac{\left(a+1.75\;L^{*}\right)}{\left(5.465{\;L}^{*}+a^{*}-3.012{\;b}^{*}\right)}$$

#### 3.2.4. Determination of Bioactive Compounds and HMF by the HPLC-PDA Method

The HPLC-PDA method was previously described by Sokół-Łętowska et al. [45]. The quantification analysis was performed using a Dionex (Germering, Germany) system, equipped with the diode array detector model Ultimate 3000, quaternary pump LPG-3400A, autosampler EWPS-3000SI, thermostated column compartment TCC-3000SD, and controlled by Chromeleon 7.2 software (Thermo Scientific Dionex, Sunnyvale, CA, USA). The Cadenza Imtakt column CD-C18 (75 × 4.6 mm, 5 μm) was used with a guard column. The column was operated at 30 °C. The mobile phase was composed of solvent A (4.5% aq. formic acid, *v*/*v*) and solvent B (100% acetonitrile). The gradient elution was as follows: 0–1 min 5% B in A, 1–20 min 25% B in A, 20–26 min 100% B, 26–30 min 5% B in A. The flow rate was 1 mL/min. Loganic acid (LA) was detected at 245 nm, gallic acid (GA) and hydroxymethylfurfural (5-hydroxymethyl-2-furfural) (HMF) at 280 nm, and ellagic acid (EA) at 254 nm. The analyses were run in three replications and the results were expressed as mg per 100 g dry weight (dw) of stones. The chemical formulas of the determined components are presented in Figure S1 and an example chromatograms from cornelian cherry stones is presented in Figures S2 and S3.

#### 3.2.5. Determination of Total Phenolic Content and Antioxidant Capacity

The total phenolic content (TPC) and the antioxidant capacity (ABTS and FRAP assay) were determined according to the procedure described by Przybylska et al. [13] and were performed using a Synergy™ H1 microplate reader (BioTek, Winooski, VT, USA). All tests were performed in four replications.

The total phenolic content (TPC) of the stone extracts was determined using the Folin–Ciocalteu reagent. Diluted stone extract (5 μL) was mixed with 10 μL of Folin–Ciocalteu reagent and 100 μL of H_2_O, and after 3 min, 50 μL of 10% sodium carbonate was also added. The mixture was shaken automatically for 30 s. The absorbance of the resulting blue color was measured at 765 nm after 1 h of incubation at room temperature in the dark. The results were calculated as mg of gallic acid equivalent (GAE) per 100 g dry weight (dw) of stones.

The ABTS^+^ (2,2′-azino-bis (3-ethyl benzothiazoline-6-sulfonic acid) solution was prepared with an absorbance of 1.000 ± 0.02 at a wavelength of 734 nm. Then, diluted stone extract (10 μL) was mixed with 200 μL of prepared ABTS^+^ solution. The mixture was shaken automatically and the absorbance reading was taken 6 min later at 734 nm.

The FRAP reagent was prepared by mixing acetate buffer (300 M, pH 3.6), a solution of 2,4,6-Tris(2-pyridyl)-s-triazine (TPTZ) in 40 mmol HCl, and a solution of FeCl_3_ × 6H_2_O in a volume ratio of 10:1:1, *v:v:v*. Then, diluted stone extract (10 μL) was mixed with 200 μL of prepared FRAP reagent. The mixture was shaken automatically and the absorbance reading was taken 10 min later at 593 nm.

The results of the ABTS and FRAP assay were expressed as µmol trolox equivalent (TE) per 1 g dry weight (dw) of stones.

#### 3.2.6. Statistical Analysis

One-way analysis of variance (roasting parameters of stones) was used to analyze the obtained data. Differences between mean values were determined using Duncan’s test at *p* ≤ 0.05. For changes in the assessed features at individual temperatures, regression analysis was performed with roasting time as the independent variable. The dynamics of changes within each temperature was described by determining regression equations in the form of a linear function (y = ax + b) or a quadratic function (y = ax^2^ + bx + c), and the degree of model fit was determined using the coefficient of determination R^2^. All analyses were conducted at least in two replications. In addition, a principal component analysis (PCA) was conducted to compare samples and features.

All statistical computations were performed using Statistica ver. 13.3 data analysis software system (StatSoft Polska Sp. z o.o. 2025. www.statsoft.pl/en/, accessed on 26 June 2025).

## 4. Conclusions

Roasting of the stones under different time–temperature conditions proceeded with different dynamics, which provided the obtained final products with different colors, contents of biologically active compounds, HMF values, and antioxidant capacities. Regression analysis showed a different nature of changes during the roasting process at 160 °C than at other temperatures, where the trends were mostly linear. Both the increase in time and temperature of roasting increased the weight loss during roasting of the stones while reducing their moisture content. The intensification of the roasting process results in dark, brownish-black colored stones, lacking the typical pink-yellow hue of unroasted stones. At the initial stages and at lower process temperatures, the contents of monomers LA, GA, and EA increased as a result of the hydrolysis of tannins, while the application of longer roasting times (40 and 50 min) and higher roasting temperatures (200–220 °C) significantly decreased these contents. Similar changes were observed in the levels of total polyphenols and antioxidative capacity, especially in the methanolic extracts. HMF content increased with increasing process intensity, but the intensity of the roasting process resulted in faster degradation. During extraction aimed at the evaluation of bioactive compounds, the use of methanol, especially at a high concentration (80%), is more advantageous than water, as it promotes more reliable results, i.e., higher contents of the compounds determined and higher antioxidant potential. In the case of unroasted stones, the type of extraction solvent does not play such a significant role as in the case of roasted stones. Similar properties and chemical compositions of the stones can be obtained by roasting under different time–temperature conditions. However, in order to maintain the highest concentration of bioactive compounds in the product, the process temperature of 160–180 °C and a time of 30–40 min were adopted as optimal conditions.

## Figures and Tables

**Table 1:** Contents of loganic acid, gallic acid, ellagic acid, and HMF in unroasted and roasted cornelian cherry stones (*Cornus mas* L.).

Roasting Parameters		Loganic Acid [mg/100 g]			Gallic Acid [mg/100 g]			Elagic Acid [mg/100 g]			HMF [mg/100 g]		
Temperature [°C]	Time [min]												
Solvent		Water	Methanol		Water	Methanol		Water	Methanol		Water	Methanol	
			50%	80%		50%	80%		50%	80%		50%	80%
Unroasted		185.44 ± 15.26 ^a^*^; A^**	137.27 ± 4.51 ^e; B^	120.84 ± 8.19 ^d; B^	182.15 ± 61.56 ^f; A^	158.46 ± 3.47 ^g; A^	152.72 ± 25.39 ^k; A^	204.32 ± 16.15 ^f; B^	182.00 ± 8.94 ^k; B^	362.73 ± 98.40 ^j; A^	nd	nd	nd
160	20	139.93 ±3.68 ^c; B^	144.88 ± 5.22 ^e; AB^	157.85 ± 2.53 ^c; A^	245.89 ± 6.78 ^e; B^	419.79 ± 52.94 ^e; A^	510.33 ± 3.72 ^h; A^	653.68 ± 0.65 ^e; B^	717.83 ± 41.04 ^j; B^	1685.59 ± 27.76 ^i; A^	48.74 ± 1.36 ^h; C^	61.99 ± 4.09 ^j; B^	81.84 ± 0.47 ^e; A^
	30	178.10 ±1.22 ^a; C^	188.95 ± 2.22 ^a; B^	210.06 ± 2.89 ^ab; A^	600.19 ± 4.00 ^b; B^	834.10 ± 14.62 ^b; B^	873.63 ± 35.49 ^e; A^	1574.53 ± 73.62 ^ab; C^	2584.16 ± 82.58 ^gh; B^	5007.34 ± 119.97 ^g; A^	164.49 ± 0.31 ^b; C^	187.84 ± 0.04 ^c; B^	223.10 ± 25.77 ^a; A^
	40	158.59 ± 1.09 ^b; C^	183.69 ± 0.89 ^a; B^	204.58 ± 5.83 ^a; A^	705.78 ± 0.11 ^a; C^	905.16 ± 11.80 ^a; B^	977.45 ± 17.81 ^d; A^	1471.13 ± 15.88 ^b; C^	4369.54 ± 81.82 ^f; B^	7111.10 ± 352.69 ^f; A^	149.26 ± 0.21 ^c; B^	196 69 ± 4.25 ^b; A^	199.75± 6.62 ^b; A^
	50	123.15 ± 2.4 ^d; B^	151.11 ± 0.13 ^c; A^	155.04 ± 2.11 ^c; A^	722.92 ± 4.42 ^a; C^	901.50 ± 18.09 ^a; B^	1201.55 ± 14.37 ^a; A^	1252.14 ± 0.82 ^c; C^	5855.42 ± 39.75 ^a; B^	8731.58 ± 92.47 ^de; A^	133.04 ± 3.43 ^d; B^	159.03 ± 5.05 ^e; A^	165.99 ± 0.59 ^c; A^
180	20	173.52 ±3.19 ^ab; C^	189.56 ± 3.25 ^a; B^	212.54 ± 5.51 ^a; A^	579.86 ± 3.43 ^b; C^	824.49 ± 1.61 ^b; B^	891.79 ± 1.02 ^e; A^	1631.29 ± 51.83 ^a; B^	2328.62 ± 127.98 ^h; A^	2467.13 ± 219.04 ^hi; A^	147.27 ± 4.59 ^c; C^	172.57 ± 3.52 ^d; B^	188.12 ± 1.05 ^b; A^
	30	121.82 ± 0.45 ^d; C^	145.36 ± 3.11 ^d; B^	152.61 ± 084 ^c; A^	723.91 ± 4.94 ^a; C^	903.55 ± 27.79 ^a; B^	1171.85 ± 7.60 ^ab; A^	1087.13 ± 51.84 ^d; C^	4822.74. ± 186.94 ^cd; B^	8043.72 ± 629.74 ^ef; A^	170.23 ± 0.16 ^b; B^	198.16 ± 9.65 ^b; A^	198.28 ± 9.39 ^b; A^
	40	77.48 ± 1.69 ^e; A^	73.42 ± 0.38 ^g; B^	70.08 ± 0.23 ^f; C^	669.67 ± 5.33 ^a; C^	806.96 ± 16.66 ^b; B^	1043.56 ± 23.90 ^c; A^	311.88 ± 0.01 ^f; C^	5065.98 ± 77.04 ^b; B^	10381.01 ± 188.72 ^bc; A^	100.27 ± 0.60 ^f; B^	115.14 ± 5.11 ^g; A^	122.10 ± 3.25 ^d; A^
	50	49.59 ±0.10 ^f; A^	47.8 ± 0.71 ^h; A^	50.84 ± 2.82 ^g; A^	512.17 ± 15.41 ^c; C^	690.10 ± 10.61 ^c; B^	770.39 ± 19.70 ^f; A^	70.00 ± 6.18 ^g; C^	4643.29 ± 111.74 ^de; B^	11571.11 ± 59.18 ^a; A^	62.63 ± 2.70 ^g; C^	86.91 ± 1.15 ^h; A^	78.76 ± 0.52 ^e; A^
200	20	137.40 ± 12.10 ^c; B^	156.43 ± 0.13 ^b; B^	184.13 ± 1.88 ^b; A^	714.16 ± 35.96 ^a; C^	899.81 ± 17.03 ^a; B^	1136.86 ± 11.43 ^b; A^	1213.88 ± 240.17 ^c; C^	4442.17 ± 135.84 ^ef; B^	7981.32 ± 247.81 ^ef; A^	181.36 ± 12.14 ^a; B^	214.05 ± 4.47 ^a; A^	203.47 ± 0.00 ^ab; A^
	30	70.32 ± 3.07 ^e; B^	72.16 ± 0.75 ^g; B^	87.53 ± 5.88 ^e; A^	662.56 ± 8.84 ^a; C^	823.79 ± 8.82 ^b; B^	1033.77 ± 63.74 ^c; A^	196.83 ± 7.45 ^f; C^	5130.71 ± 108.77 ^b; B^	11586.11 ± 770.74 ^a; A^	111.30 ± 0.08 ^e; B^	132.29 ± 5.31 ^f; A^	135.51 ± 9.82 ^d; A^
	40	42.05 ± 1.05 ^fg; B^	41.11 ± 1.47 ^i; B^	52.74 ± 1.07 ^g; A^	405.11 ± 2.94 ^d; C^	551.49 ± 23.00 ^d; B^	609.31 ± 5.36 ^g; A^	45.69 ± 3.75 ^g; C^	2735.57 ± 205.5 ^g; B^	11072.61 ± 63.73 ^ab; A^	62.64 ± 0.28 ^g; B^	82.65 ± 1.96 ^hi; A^	44.93 ± 31.77 ^f; A^
	50	18.23 ± 0.09 ^hi; AB^	15.38 ± 0.91 ^k; B^	23.01 ± 2.70 ^i; A^	13383 ± 0.01 ^f; C^	203.93 ± 4.48 ^f; B^	229.33 ±1.58 ^j; A^	21.42 ± 7.75 ^g; C^	2801.01 ±50.58 ^g; B^	9800.27 ± 545.69 ^cd; A^	24.29 ± 1.33 ^i; B^	40.0 ± 0.08 ^k; A^	0.00± 0.00 ^g; A^
220	20	76.09 ±0.50 ^e; C^	85.30 ± 0.19 ^f; B^	96.31 ± 2.41 ^e: A^	682.30 ± 15.03 ^a; C^	824 ± 1.39 ^b; B^	883.80 ± 10.41 ^e; A^	220.22 ± 38.89 ^f; C^	4895.07 ± 2.10 ^bc; B^	9700.54 ± 678.54 ^cd; A^	132.52 ± 0.82 ^d; B^	153.05 ± 0.30 ^e; A^	155.31 ± 3.10 ^c; A^
	30	29.74 ±2.12 ^gh; B^	31.30 ± 0.01 ^j; B^	38.22 ± 2.81 ^h; A^	279.65 ± 4.33 ^e; C^	386.30 ± 0.28 ^e; B^	421.02 ± 3.92 ^i; A^	46.36 ± 4.10 ^g; C^	2809.72 ± 156.44 ^g; B^	4755.82 ± 261.44 ^g; A^	54.84 ± 1.40 ^h; B^	75.5 ± 0.17 ^i; A^	78.03 ± 1.15 ^e; A^
	40	11.76 ± 1.27 ^ij; C^	13.39 ± 0.15 ^k; B^	17.16 ± 4.94 ^i; A^	60.24 ± 7.70 ^g; C^	96.10 ± 2.90 ^h; B^	123.56 ± 0.73 ^k; A^	28.32 ± 6.57 ^g; C^	1446.77 ± 125.13 ^i; B^	3155.82 ± 433.42 ^h; A^	15.57 ± 2.34 ^j; B^	33.35 ± 2.45 ^k; A^	39.04 ± 0.75 ^f; A^
	50	4.91 ± 0.07 ^j; C^	12.37 ± 0.15 ^k; B^	18.30 ± 0.24 ^i; A^	0.00 ± 0.00 ^h; B^	0.15 ± 0.09 ^i; B^	6.93 ± 2.22 ^l; A^	17.47 ± 2.40 ^g; C^	658.41 ± 4.60 ^j; B^	1978.86 ± 121.40 ^i; A^	0.00 ± 0.00 ^k; B^	5.52 ± 0.75 ^l; A^	0.00 ± 0.00 ^g; B^

Homogeneous groups determined by Duncan test (*p* ≤ 0.05), * lower-case letter represents homogenous group by roasting condition; ** capital letter represents homogenous group by solvent type; nd—not detected.

**Table 2:** Total polyphenol content and antioxidant potential (ABTS, FRAP) in unroasted and roasted cornelian cherry stones (*Cornus mas* L.).

Roasting Parameters		Total Phenolic Content [mg GEA/g]			ABTS [µmol TE/g]			FRAP [µmol TE/g]		
Temperature [°C]	Time [min]									
Solvent		Water	Methanol		Water	Methanol		Water	Methanol	
			50%	80%		50%	80%		50%	80%
Unroasted		3939.10 ± 66.66 ^a; A^	3438.7 ± 156.63 ^cd; A^	4159.5 ± 135.16 ^ab; A^	353.20 ± 0.55 ^a; A^	360.3 ± 0.39 ^e; A^	421.1 ± 4.26 ^j; A^	273.7 ± 1.01 ^g; A^	286.0 ± 1.16 ^c; A^	334.1 ± 4.58 ^i; A^
160	20	2445.38 ± 166.14 ^b; C^	3553.50 ± 150.97 ^bc; B^	3952.00 ± 169.55 ^bc; A^	289.43 ± 82.49 ^ab; C^	509.26 ± 37.02 ^b; B^	1214.84 ± 4.27 ^a; A^	933.47 ± 38.78 ^a; A^	339.81 ± 20.90 ^b; B^	792.60 ± 12.08 ^bc; B^
	30	1148.76 ± 14.80 ^d; C^	3896.00 ± 84.76 ^ab; B^	4299.50 ± 71.18 ^ab; A^	238.34 ± 58.82 ^bc; C^	522.73 ± 5.52 ^b; B^	1173.31 ± 0.36 ^ab; A^	829.68 ± 9.99 ^b; A^	357.87 ± 19.51 ^b; B^	826.76 ± 3.72 ^ab; A^
	40	670.56 ± 85.99 ^e; C^	3787.00 ± 235.86 ^abc; B^	4402.00 ± 127.23 ^a; A^	173.84 ± 40.58 ^cd; C^	515.18 ± 3.03 ^b; B^	1205.28 ± 24.92 ^a; A^	768.92 ± 27.17 ^c; A^	355.58 ± 16.72 ^b; B^	817.57 ± 4.64 ^ab; A^
	50	159.84 ± 40.22 ^fgh; C^	2769.90 ± 613.83 ^f; B^	4196.00 ± 145.40 ^ab; A^	140.69 ± 5.54 ^de; C^	442.32 ± 21.36 ^c; B^	1055.78 ± 43.42 ^cd; A^	655.11 ± 12.08 ^d; B^	296.62 ± 9.99 ^c; C^	793.92 ± 10.22 ^bc; A^
180	20	1691.05 ± 55.20 ^c; C^	3985.00 ± 285.66 ^a; B^	4232.00 ± 113.49 ^ab; A^	171.74 ± 45.91 ^cd; C^	582.63 ± 18.33 ^a; B^	1233.47 ± 35.59 ^a; A^	432.90 ± 4.53 ^e; B^	385.63 ± 8.59 ^a; C^	878.33 ± 4.18 ^a; A^
	30	1115.25 ± 134.50 ^d; C^	3044.30 ± 181.31 ^ef; B^	3989.5 ± 146.47 ^bc; A^	111.96 ± 12.19 ^def; C^	415.77 ± 26.16 ^cd; B^	1024.07 ± 47.69 ^cd; A^	308.10 ± 1.74 ^f; B^	283.98 ± 17.19 ^c; B^	705.57 ± 63.63 ^de; A^
	40	665.75 ± 112.37 ^f; C^	2142.10 ± 92.42 ^g; B^	3730.00 ±146.07 ^cd; A^	55.02 ± 5.88 ^fg; C^	302.64 ± 13.88 ^f; B^	942.02 ± 15.66 ^de; A^	217.04 ± 2.55 ^h; C^	227.65 ± 1.16 ^d; B^	626.41 ± 2.79 ^fg; A^
	50	205.27 ± 43.33 ^fgh; C^	1615.45 ± 81.50 ^hi; B^	3105.5 ± 95.37 ^fg; A^	41.59 ± 0.40 ^fg; C^	245.13 ± 20.11 ^g; B^	756.28 ± 14.95 ^gh; A^	151.02 ± 5.34 ^ij; B^	204.16 ± 12.08 ^e; B^	461.21 ± 28.33 ^h; A^
200	20	694.12 ± 31.00 ^e; C^	3178.00 ± 58.96 ^de; B^	4100.00 ± 154.69 ^ab; A^	66.43 ± 36.31 ^efg; C^	394.94 ± 8.90 ^d; B^	1087.99 ± 66.91 ^bc; A^	174.86 ± 21.95 ^i; C^	299.89 ± 3.37 ^c; B^	724.29 ± 39.02 ^cd; A^
	30	430.05 ± 58.41 ^efg; C^	2296.65 ± 132.68 ^g; B^	3494.00 ± 221.18 ^de; A^	56.45 ± 7.07 ^fg; C^	299.24 ± 2.05 ^f; B^	881.62 ± 12.81 ^ef; A^	112.05 ± 3.25 ^k; C^	232.31 ± 0.23 ^d; B^	637.83 ± 70.52 ^ef; A^
	40	206.10 ± 91.29 ^fgh; C^	1279.85 ± 99.47 ^ij; B^	2927.00 ± 125.43 ^fg; A^	37.52 ± 2.34 ^fg; C^	203.16 ± 3.92 ^h; B^	650.83 ± 3.20 ^hi; A^	62.70 ± 0.70 ^l; C^	158.98 ± 1.97 ^f; B^	560.07 ± 0.93 ^fg; A^
	50	27.20 ± 18.11 ^h; C^	1109.25 ± 81.79 ^jk; B^	2484.7 ± 113.10 ^hi; A^	30.04 ± 1.45 ^g; C^	145.22 ± 2.76 ^ij; B^	469.11 ± 117.81 ^j; A^	39.46 ± 6.62 mL^; B^	115.06 ± 8.13 ^g; B^	273.01 ± 73.9 ^i; A^
220	20	494.14 ± 133.49 ^efg; C^	1938.40 ± 226.20 ^gh; B^	3237.50 ± 154.19 ^ef; A^	69.46 ± 16.39 ^efg; C^	304.65 ± 4.18 ^f; B^	821.72 ± 4.98 ^fg; A^	127.98 ± 7.55 ^jk; C^	243.64 ± 0.46 ^d; B^	556.13 ± 5.57 ^h; A^
	30	265.89 ± 33.93 ^fgh; C^	1089.95 ± 68.75 ^jk; B^	2783.1 ± 167.17 ^gh; A^	40.21 ± 1.09 ^fg; C^	175.73 ± 0.18 ^hi; B^	622.89 ± 11.39 ^i; A^	52.81 ± 23.86 ^l; B^	137.80 ± 1.51 ^f; B^	413.26 ± 41.34 ^h; A^
	40	99.50 ± 33.00 ^gh; C^	948.75 ± 46.51 ^jk; B^	2380.90 ± 85.25 ^i; A^	32.95 ± 15.57 ^fg; C^	131.69 ± 0.36 ^j; B^	500.07 ± 135.96 ^j; A^	36.10 ± 1.31 mL^; C^	97.48 ± 0.46 ^gh; B^	296.00 ± 10.22 ^i; A^
	50	30.58 ± 14.69 ^h; C^	864.90 ± 65.56 ^k; B^	2237.80 ± 126.82 ^i; A^	23.11 ± 5.56 ^g; C^	100.16 ± 2.40 ^k; B^	448.98 ± 21.0 ^j; A^	19.76 ± 1.39 ^m; C^	84.10 ± 2.67 ^h; B^	261.52 ± 6.97 ^i; A^

Homogeneous groups determined by Duncan test (*p* ≤ 0.05), lower-case letter represents homogenous group by roasting condition; capital letter represents homogenous group by solvent type.

## Data Availability

The original results presented in this study are included in the article. Further inquiries can be directed to the corresponding author.

## Supplementary Materials

The following supporting information can be downloaded at: https://www.mdpi.com/article/10.3390/molecules30142900/s1, Table S1: Colour characteristics (Δ*E,* White Index (WI), Browning index (BI)) of unroasted and roasted cornelian cherry stones and their changes during roasting; Table S2: Characteristics of the roasting process (LA, GA, EA, and HMF content) at individual temperatures depending on time; Table S3: Characteristics of the roasting process (total phenolic content, ABTS, FRAP) at individual temperatures depending on time; Figure S1. Structural formulas of bioactive compounds detected in cornelian cherry stones: loganic acid (a), gallic acid (b), HMF (c), and ellagic acid (d). Formulas were developed in ChemDraw Ultra version 12.0.2; Figure S2. Cornelian cherry stone chromatograms examples with marked of bioactive compounds: loganic acid (LA) and ellagic acid (EA); Figure S3. Cornelian cherry stone chromatograms examples with marked of bioactive compounds: gallic acid (GA) and HMF.
