# Supplementary material for: Selection of Roasting Conditions in the Valorization Process of Cornelian Cherry Stones
Source: Molecules. 2025 Jul 8;30(14):2900. doi: 10.3390/molecules30142900 (PMC12298863; doi:10.3390/molecules30142900)
Supplement: Supplementary file 1 [file molecules-30-02900-s001.zip › molecules-3686712-supplementary.pdf]

Selection of roasting conditions in the valorization process of Cornelian cherry stones

Table S1. Colour characteristics (*ΔE,* White Index (WI), Browning index (BI)) of unroasted and roasted cornelian cherry stones and their changes during roasting.

Feature	Roasting temperature [°C]	Multiple R^2	Regression equation
ΔE	160	0.972	0.874x-9.916
	180	0.906	0.756x+5.650
	200	0.944	0.486x+22.158
	220	0.958	0.394x+31.221
White Index (WI).	160	0.953	-0.524x+56.460
	180	0.949	-0.463x+47.202
	200<br>220	0.972<br>0.918	-0.341x+38.269<br>-0.272x+31.975
Browning index (BI))	160	0.992<br>0.284	$-0.054x^2+3.452x+31.982$<br>-0.304 x+91.011
	180	0.998<br>0.824	$0.046x^2-4.132x+153.392$<br>-0.897x+102.562
	200	0.891<br>0.811	$0.023x^2-2.237x+11.839$<br>-0.648x+86.857
	220	0.928	-1.028x+86.436

Selection of roasting conditions in the valorization process of Cornelian cherry stones

Table S2. Characteristics of the roasting process (LA, GA, EA, and HMF content) at individual temperatures depending on time.

Feature	Solvent	Roasting temperature [°C]	Multiple R^2	Regression equation
Loganic acid [mg/100g]	Water	160	0.130<br>0.940	$-0.669x+173.091$<br>$-0.186x^2+12.389x-32.097$
		180	0.984	$-4.131x+249.949$
		200	0.925	$-3.858x+202.026$
		220	0.868	$-2.315x+111.649$
	50 % methanol	160	0.006<br>0.973	$0.013x+1.638$<br>$-0.192x^2+13.548x-48.323$
		180	0.971	$-4.942x+286.760$
		200	0.914	$-4.542x+230.232$
		220	0.795	$-2.367x+118.437$
	80 % methanol	160	0.002<br>0.986	$0.011+1.844$<br>$-0.257x^2+17.873x-97.134$
		180	0.953	$-5.677x+320.196$
		200	0.912	$-5.182x+268.211$
		220	0.782	$-2.551x+131.776$
Gallic acid [mg/100g]	Water	160	0.799	$15.367x+30.853$
		180	0.124<br>0.977	$-0.257x+6.857$<br>$-0.754x^2+50.198x+117.801$
		200	0.932	$-19.984x+1178.369$
		220	0.897	$-22.663x+1048.753$
	50 % methanol	160	0.701	$15.162x+234.471$
		180	0.523<br>0.925	$-4.998x+981.190$<br>$-0.490x^2+29.288+442.417$
		200	0.934	$-23.599x+1445.735$
		220	0.929	$-27.606x+1292.769$
	80 % methanol	160	0.949	$+21.785x+128.022$
		180	0.132<br>0.958	$-0.492x+10.920$<br>$-1.381x^2+91.725x-377.463$
		200	0.947	$-31.460x+1853.179$
		220	0.935	$-29.281x+1383.645$
Ellagic acid [mg/100g]	Water	160	0.300<br>0.914	$1.692x+8.149$<br>$-2.850x^2+216.391x-2488.870$
		180	0.965	$-54.593x+2685.788$
		200	0.697	$-37.285x+1674.437$
		220	0.697	$-6.263x+297.230$
	50 % methanol	160	0.907	$171.981x-2637.605$
		180	0.528	$71.902x+1698.329$
		200	0.616	$-73.186x+6338.882$
		220	0.957	$-140.719x+7377.413$
	80 % methanol	160	0.968	$232.417x+-2501.209$
		180	0.889	$296.490x-2261.490$
		200	0.141<br>0.825	$6.087x+86.837$<br>$-12.194x^2+902.984x-5032.894$
		220	0.867	$-247.650x+13565.540$
HMF [mg/100g]	Water	160	0.352<br>0.895	$0.238x+645$<br>$-0.330x^2+25.472x-322.228$
		180	0.753	$-2.239x+233.461$
		200	0.976	$-5.199x+276.851$
		220	0.907	$-4.3684x+203.626$
	50 % methanol	160	0.394<br>0.975	$0.303x+0.754$<br>$-0.411x^2+31.820x-407.319$
		180	0.731	$-3.400x+262.205$
		200	0.974	$-5.718x+317.374$
		220	0.948	$-4.847x+236.514$
	80 % methanol	160	0.229<br>0.870	$0.229x+1.104$<br>$-0.438x^2+32.920x-393.835$
		180	0.847	$-4.043x+288.317$
		200	0.986	$-7.104x+345.726$
		220	0.966	$-5.049x+244.821$

Selection of roasting conditions in the valorization process of Cornelian cherry stones

Table S3. Characteristics of the roasting process (total phenolic content, ABTS, FRAP) at individual temperatures depending on time.

Feature	Solvent	Roasting temperature [°C]	Multiple R^2	Regression equation
Total phenolic content [mg GEA/g]	Water	160	0.924	-73.348x+3673.328
		180	0.972	-49.069x+2636.727
		200	0.952	-22.247x+1118.015
		220	0.832	-15.571x+767.507
	50 % methanol	160	0.261	-24.598x+4362.530
		180	0.953	-80.109x+5500.510
		200	0.927	-72.231x+4494.005
		220	0.720	-33.607x+2386.495
	80 % methanol	160	0.204	8.345x+3920.300
		180	0.873	-36.390x+5037.900
		200	0.941	-54.130x+5145.945
		220	0.867	-34.014x+3850.285
ABTS [µmol TE/g]	Water	160	0.630	-5.107x+389.326
		180	0.850	-4.474x+251.665
		200	0.973	-1.281x+92.441
		220	0.730	-1.463x+92.644
	50 % methanol	160	0.429<br>0.796	-2.084x+570.310<br>$-0.216x^2+13.023x+332.913$
		180	0.943	-11.256x+780.514
		200	0.887	-8.453x+556.485
		220	0.891	-6.575x+408.189
	80 % methanol	160	0.571	-4.452x+1318.131
		180	0.960	-15.136x+1518.720
		200	0.957	-20.874x+1502.990
		220	0.837	-12.410x+1032.776
FRAP [µmol TE/g]	Water	160	0.959	-8.958x+1110.330
		180	0.980	-9.367x+605.113
		200	0.940	-4.556x+256.710
		220	0.816	-3.440x+178.901
	50 % methanol	160	0.288<br>0.780	-1.319x+383.624<br>$-0.193x^2+12.160x+171.821$
		180	0.910	-6.007x+485.604
		200	0.988	-6.278x+421.293
		220	0.859	-5.189x+322.383
	80 % methanol	160	0.572<br>0.819	-15.136x+1518.720<br>$-0.145x^2+10.063x+650.586$
		180	0.956	-13.305x+1133.557
		200	0.501	-14.816x+1079.857
		220	0.925	-10.011x+732.105

a) 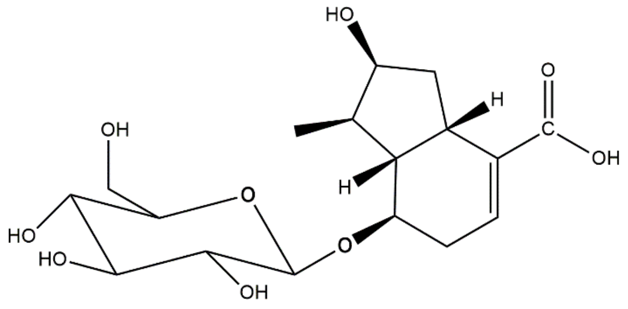

b) 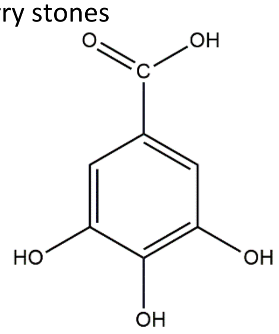

c) 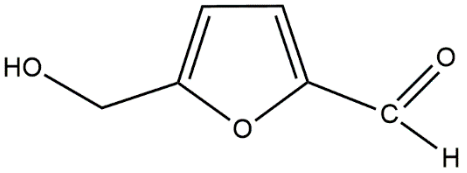

d) 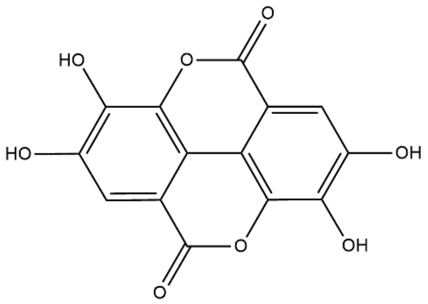

Figures S1. Structural formulas of bioactive compounds detected in cornelian cherry stone: loganic acid (a), gallic acid (b), HMF (c) and ellagic acid (d). Formulas was developed in ChemDraw Ultra version 12.0.2.

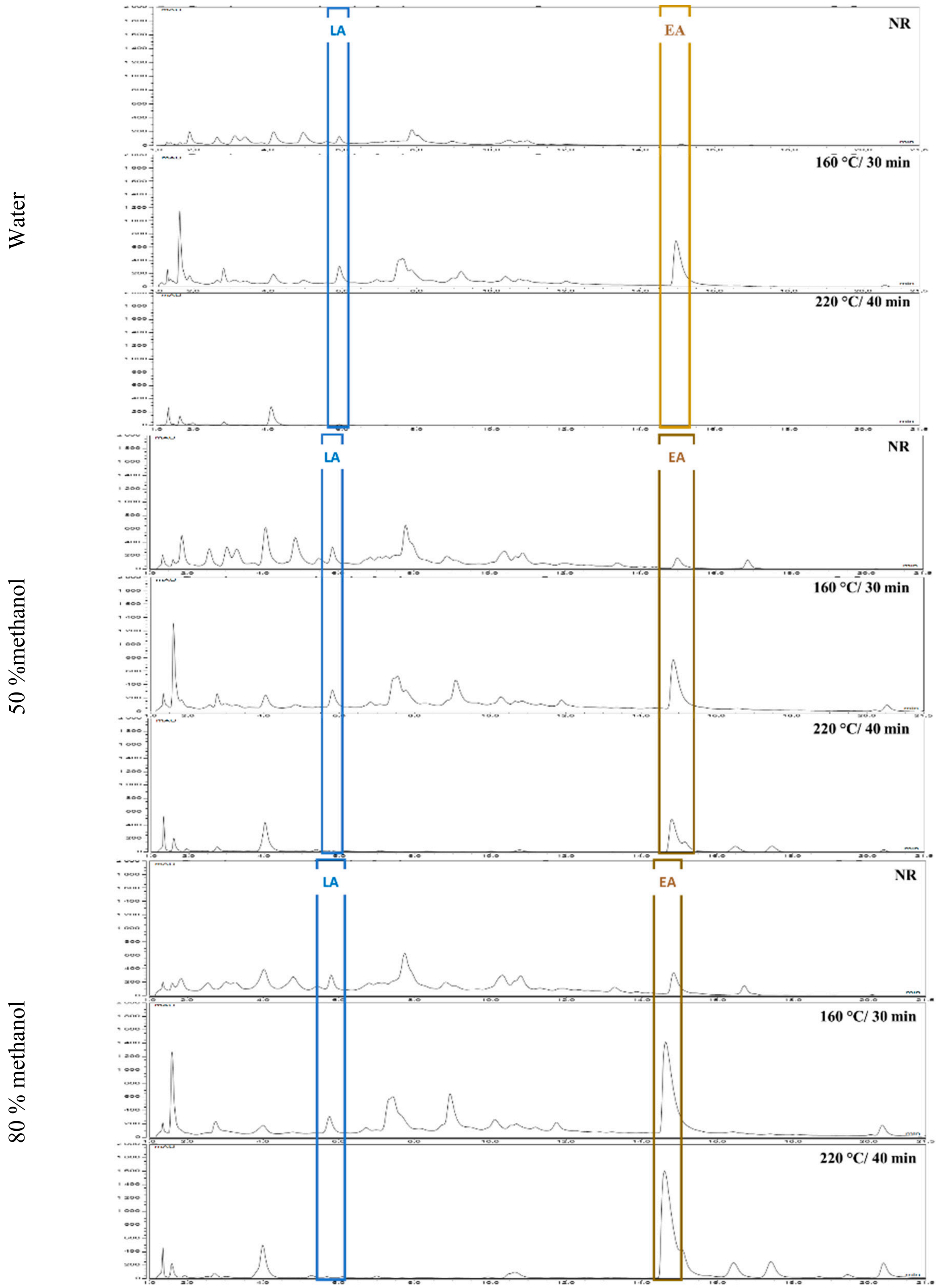

Figure S2. Cornelian cherry stone chromatograms examples with marked of bioactive compounds: loganic acid (LA) and ellagic acid (EA).

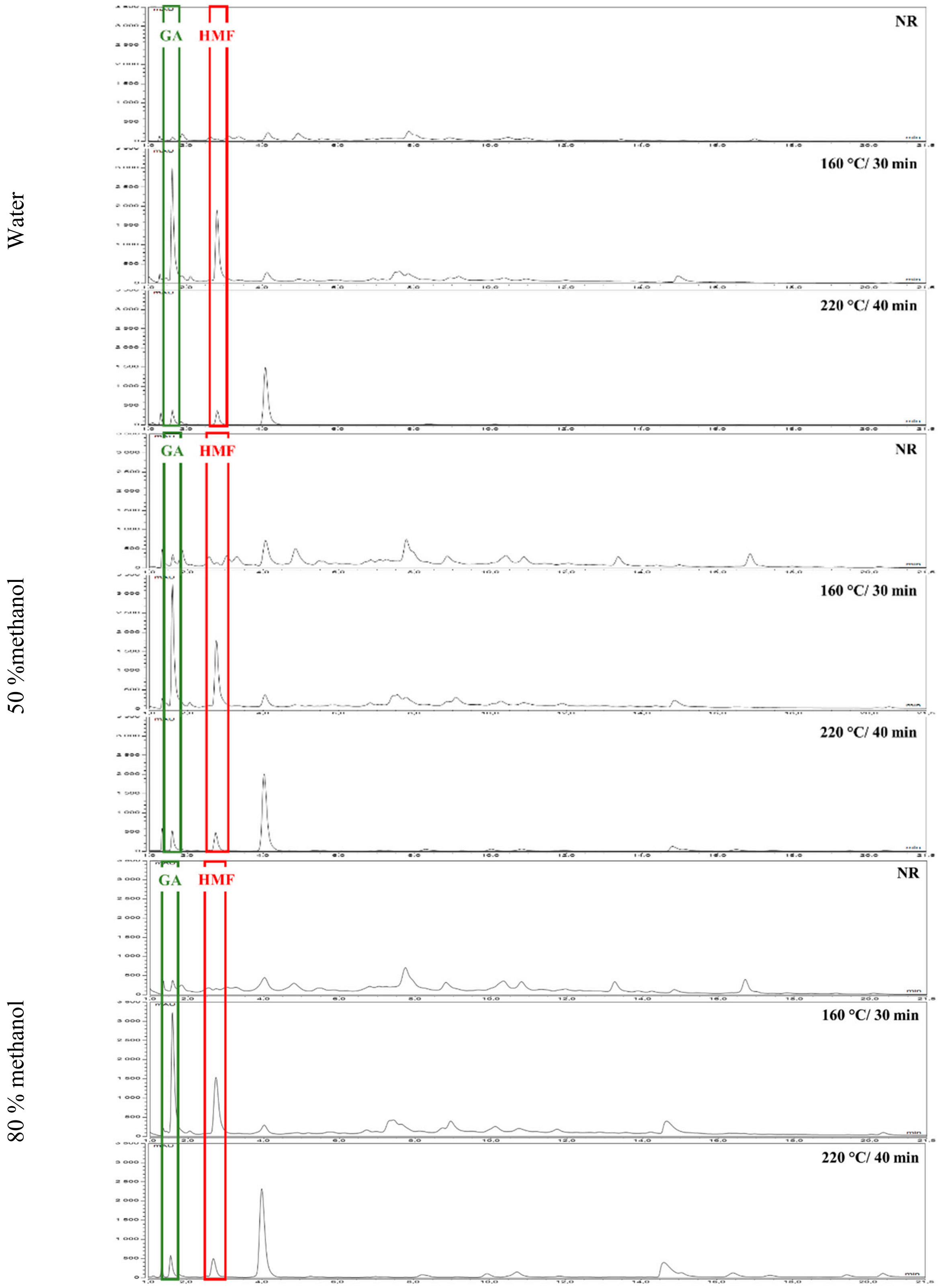

Figure S3. Cornelian cherry stone chromatograms examples with marked of bioactive compounds gallic acid (GA) and HMF.
